# Proteomic Approach to Reveal the Regulatory Function of Aconitase AcnA in Oxidative Stress Response in the Antibiotic Producer *Streptomyces viridochromogenes* Tü494

**DOI:** 10.1371/journal.pone.0087905

**Published:** 2014-02-03

**Authors:** Ewelina Michta, Wei Ding, Shaochun Zhu, Kai Blin, Hongqiang Ruan, Rui Wang, Wolfgang Wohlleben, Yvonne Mast

**Affiliations:** 1 Department of Microbiology/Biotechnology, Interfaculty Institute of Microbiology and Infection Medicine, Eberhard Karls University of Tübingen, Tübingen, Germany; 2 Shanghai Applied Protein Technology Co., Ltd., Shanghai, China; 3 Key Laboratory of Synthetic Biology, Institute of Plant Physiology and Ecology, Shanghai Institutes for Biological Sciences, Chinese Academy of Sciences, Shanghai, China; University of South Florida College of Medicine, United States of America

## Abstract

The aconitase AcnA from the phosphinothricin tripeptide producing strain *Streptomyces viridochromogenes* Tü494 is a bifunctional protein: under iron-sufficiency conditions AcnA functions as an enzyme of the tricarboxylic acid cycle, whereas under iron depletion it is a regulator of iron metabolism and oxidative stress response. As a member of the family of iron regulatory proteins (IRP), AcnA binds to characteristic iron responsive element (IRE) binding motifs and post-transcriptionally controls the expression of respective target genes. A *S. viridochromogenes* aconitase mutant (MacnA) has previously been shown to be highly sensitive to oxidative stress. In the present paper, we performed a comparative proteomic approach with the *S. viridochromogenes* wild-type and the MacnA mutant strain under oxidative stress conditions to identify proteins that are under control of the AcnA-mediated regulation. We identified up to 90 differentially expressed proteins in both strains. *In silico* analysis of the corresponding gene sequences revealed the presence of IRE motifs on some of the respective target mRNAs. From this proteome study we have *in vivo* evidences for a direct AcnA-mediated regulation upon oxidative stress.

## Introduction

Streptomyces are gram-positive soil bacteria that undergo a complex life cycle where morphological differentiation is strongly coordinated with secondary metabolites production [1]. *Streptomyces viridochromogenes* Tü494 is the producer of the herbicide antibiotic phosphinothricin tripeptide (PTT). In this strain the function of the protein AcnA was analyzed in detail previously, because it represents a nodal point between primary and secondary metabolism [2]–[4]. It has been shown before that an *S. viridochromogenes* aconitase mutant (MacnA) is unable to form any aerial mycelium, spores nor PTT [3]. Furthermore, this mutant is highly sensitive to oxidative stress [5]. As reported for *Escherichia coli* aconitase A this oxidative stress hypersensitivity is not due to the lack of a citric acid cycle function *per se*, but to some extent may be associated with the loss of the regulatory function of AcnA [6]. The AcnA enzymes from *E. coli* and *S. viridochromogenes* both belong to the same family of iron regulatory proteins (IRPs).

One of the best studied aconitases is the human cytosolic iron regulatory protein 1 (IRP1). Under normal physiological conditions IRP1 functions as a tricarboxylic acid cycle (TCA) enzyme and converts citrate to isocitrate. However, under iron deficiency or oxidative stress conditions, the 4Fe-4S cluster in the catalytic center of IRP1 is disassembled and the protein undergoes conformational changes, resulting in the catalytically inactive apo-enzyme [7] that becomes open and accessible for the binding of sequences known as iron responsive elements (IREs) [8]. IREs form stem-loop structures at the 5′ or 3′ end of untranslated regions (UTRs) of mRNA transcripts such as that of the iron-storage protein ferritin. For IRP1 it has been shown that the binding to the 5′ UTR inhibits translation by blocking the mRNA-ribosome complex formation, while binding at the 3′ UTR protects the mRNA from degradation by RNases, which stabilizes the transcript and in this way stimulates translation [7]. In prokaryotic cells, there is no evidence so far that the binding of aconitase to the 5′ or 3′ ends of mRNA exerts different effects on translation.

The major regulatory role of IRP1 in eukaryotes is to control iron homeostasis by binding to mRNAs such as that of the iron-storage protein ferritin. For several bacterial species, e.g. *E. coli*, *Mycobacterium tuberculosis*, and *Bacillus subtilis*, also a stress-dependent binding of aconitase to IRE-like structures of genes involved in oxidative stress has been reported [6], [9]–[12]. Oxidative stress has a broad influence on cellular metabolism. Notably, the reactivity of H_2_O_2_ with iron in the Fenton reaction connects oxidative stress and cellular iron metabolism. In this reaction Fe^2+^ generates highly reactive hydroxyl radicals, which quickly abstract H-atoms from most organic molecules [13].

A regulatory function of aconitase in oxidative stress control is also ascribed for AcnA of *S. viridochromogenes* Tü494. In a related paper, we presented *in vitro* shift experiments that proved the binding of apo-AcnA to different IRE structures, such as that of *recA*, which is crucial for the cellular SOS response. Furthermore, by immunoblot analysis we found that RecA expression is upregulated under oxidative stress conditions in the wild-type strain (WT) but not in the aconitase mutant MacnA, which demonstrates that AcnA is a post-transcriptional regulator of RecA expression under oxidative stress [5].

In the current study we used a proteomic approach to investigate the regulatory function of AcnA under oxidative stress conditions. We also aimed to identify enzymes that are crucial for the *S. viridochromogenes* oxidative stress adaptation. Besides that we tried to explore the changes in protein expression that may be associated with the impaired defense of the aconitase mutant MacnA against free radicals.

## Materials and Methods

### Bacterial strains, plasmids and growth conditions

For preparation of cell extracts applied for proteome analyses, the *S. viridochromogenes* WT and previously described aconitase mutant strain MacnA [3] were each grown in 100 ml of S-medium at 27°C on an orbital shaker (180 rpm) in 500 ml Erlenmeyer flasks with steel springs to the mid-exponential growth phase. Because WT and MacnA mutant strains showed different growth rates, samples were taken for different time points and DNA concentration of the biomass was estimated as described before [14] to ensure that the strains were in a comparable growth status (Fig. S1). Each culture was supplemented with 0.2 mM methyl viologen dichloride hydrate (MV). MV is an inducer of oxidative stress and initiates the transition of AcnA into its regulatory active apo-form. Cultures were incubated with MV on an orbital shaker (180 rpm) for 90 min at 27°C. Subsequently, identical amounts of mycelium from each strain were harvested by centrifugation (10 min, 5000 rpm). The cell pellets were washed twice with S-medium and then used for proteome analysis. As control, MV-untreated samples were used.

### *In silico* analysis for identification of IRE sequences

To identify putative IRE sequences of genes encoding proteins identified in the proteomic analysis, the bioinformatical tool SPIRE was used (http://www.unituebingen.de/en/32760). The SPIRE algorithm and its implementation in searching for conserved IRE-like stem-loop structures has been described previously [5].

### 2D electrophoresis

#### Sample preparation

100 mg of mycelium from each culture was ground in liquid nitrogen and then dissolved in 2D lysis buffer (8 M urea, 150 mM Tris/HCl pH 8, 4% CHAPS, 40 mM DTT). Protease inhibitor cocktail (Sigma) (sample:protease ratio = 25∶1) was added to prevent proteolytic degradation. To increase the lysis efficiency, samples were incubated for 45 min at 35°C (incubation temperature should not exceed 37°C because the urea of the lysis buffer causes protein modifications changing the molecular weight). After incubation the samples were sonicated on ice and centrifuged (45 min, 14000 g, 4°C). Protein concentrations were estimated by Bradford assay [15].

#### Protein separation

100 µg of protein from each sample was applied for analytical gels (used for image analysis and spot quantification) and 400 µg for preparative gels (used for spots excision). The total volume of each sample was made up to 250 µl with rehydratation buffer (330 mM urea, 1.3 mM CHAPS, 0.0045% bromophenol blue). The protein mixture was applied to Immobiline DryStrip strips (13 cm, pH 4–7; GE Healthcare). Isoelectric focusing (IEF) was performed with an Ettan IPGphor II apparatus (GE Healthcare) with following steps: 30 V for 12 h, 500 V for 1 h, 1000 V for 1 h, and 8000 V for 8 h. After IEF, each strip was equilibrated with a reducing equilibration buffer (6 M urea, 50 mM Tris/HCl pH 8.8, 30% glycerol, 2% SDS, 1% DTT) for 15 min followed by the incubation with an alkylating equilibration buffer (6 M urea, 50 mM Tris/HCl pH 8.8, 30% glycerol, 2% SDS, 1% iodoacetamide) for another 15 min. The strips were placed on the top of 12.5% SDS-PAGE gels and sealed with 0.5% agarose. Separation of the proteins in the second dimension occurred at 30 mA per gel in an Ettan DALT six instrument (GE Healthcare) until the bromophenol blue reached the bottom of the gel. Gels were fixed overnight in a mixture of 10% ethanol and 10% acetic acid. Analytical and preparative gels were stained with silver solution and scanned with Typhoon FLA9000 Variable Mode Imager (GE Healthcare) (Fig. S2).

#### Protein identification

To exclude artefacts caused by electrophoresis conditions, 2D analytical gels were run in triplicate. Spot detection, quantification, and matching were performed using Image Master 2D Platinum software version 5.0 according to the manufacturer's instruction (GE Healthcare). Spots were quantified calculating the relative spot volumes (ratio of individual spot volume and total volume of all spots). Significantly changed proteins between treated and control sample were defined as proteins that were more or less abundant by a ratio of 1.1. Spots of interest, identified by Image Master, were manually matched to the analytical gels and excised. The digestion in trypsin solution (Promega) was performed overnight at 37°C. The tryptic peptides were extracted with 60% acetonitrile and 0.1% trifluoroacetic acid. The dry peptide samples were reconstituted in 2 µl of standard diluent (acetonitrile to water ratio = 20∶80), spotted on a 384-well Opti-TOF stainless steel plate, covered with 0.6 µl of matrix (5 mg/ml α-cyano-4-hydroxycinnamic acid in 50% acetonitrile and 0.1% trifluoroacetic acid) and air dried. MS and MS/MS data for protein identification were obtained by using MALDI-TOF-TOF instrument (4800 proteomics analyzer; Applied Biosystems). Combined peptide mass fingerprinting and MS/MS queries were performed using the MASCOT search engine 2.2 (Matrix Science, Ltd.) embedded into GPS-Explorer Software 3.6 (Applied Biosystems) on the NCBI database where the taxonomy was set to: Bacteria (Table S1). Only proteins with the GPS Explorer protein confidence index ≥95% were assumed to be significant (p<0.05) and successfully identified. The spots that did not fulfill this criterion were excluded from the results.

## Results and Discussion

### Identification of differentially expressed proteins in *S. viridochromogenes* WT and MacnA

To investigate the potential regulatory role of aconitase in oxidative stress defense in *Streptomyces viridochromogenes* Tü494 and to reveal the cellular mechanisms underlying the oxygen stress sensitivity in the aconitase mutant MacnA, a proteomic comparison between the *S. viridochromogenes* wild-type (WT) strain and MacnA under oxidative stress condition was performed. WT and MacnA were grown in liquid cultures to the mid-exponential growth phase, where each culture was supplemented with methyl viologen (MV). MV is an inducer of oxidative stress and also leads to the disassembly of holo-AcnA into its regulatory active apo-AcnA form [9]. The cytoplasmic fractions of both cultures were used for proteomic analysis. Fractions that were not treated with MV served as controls. To better distinguish between significant changes related to the aconitase mutation and changes related to oxidative stress response, the up- or downregulated proteins were identified separately for the WT strain and the MacnA mutant after oxidative stress treatment. This was conducted by comparison of the two-dimensional polyacrylamide gel electrophoresis (2D-PAGE) image of the MV-treated WT strain sample with the 2D-PAGE image of the MV-untreated WT strain sample (Fig. S2 A, B). The same was done for the mutant strain MacnA (Fig. S2 C, D). Thus, whenever a protein is described below to be up- or downregulated, this is in comparison to the control sample (MV-untreated) of the respective strain.

In the WT strain, 62 protein spots with significant differences in the expression level due to oxidative stress application were detected and identified by MALDI TOF/TOF. Among them 37 were upregulated and 25 were downregulated. In the MacnA strain, 27 proteins were differentially expressed as a consequence of oxidative stress treatment and identified by MALDI TOF/TOF: 16 among them were upregulated and 11 were downregulated. Some of the identified proteins, which are discussed below, occurred as multiple spots on the gel, which may be due to post-translation modifications.

The majority of the differentially regulated proteins identified in the WT strain were different from those detected in MacnA. This is because the respective 2D gels were not matched with each other by the proteome imaging program because then it would not be possible to distinguish between influence of oxidative stress from influence of *acnA* mutation. Also in other proteomic studies, where oxidative stress was investigated in different strains, it has been observed that just very few (if any) of the same proteins were identified in all analyzed gels [16], [17]. Proteins that were identified as up- or downregulated in one strain but their expression was not, or differentially, changed in the respective other strain after oxidative stress application, were considered to be affected by AcnA. The proteins that were similarly expressed in both strains due to oxidative stress exposure were considered to be independent from AcnA regulation.

All identified proteins, could be assigned to different metabolic pathways such as: carbon metabolism, fatty acid metabolism, amino acid biosynthesis, phosphate uptake and phosphate metabolism, protein synthesis and turnover, DNA synthesis, proteins involved in control of energy status, and proteins induced in response to stress conditions (Table S1). However, no polypeptides were identified, which are directly involved in iron metabolism. This corresponds to the observations from our previous analysis of *S. viridochromogenes* where nearly no IRE-like structures have been found in the UTRs of genes known to be involved in iron metabolism [5].

As expected, many of the identified proteins were related to cellular stress response and several of them were found to possess predicted IRE motifs on their respective mRNAs (Table S1 and 1), which points to a direct post-transcriptional regulation by AcnA. Especially two proteins with putative functions in cellular stress defense (tellurium resistance protein SSQG_02339 and translation elongation factor Tu SSQG_04757) were differentially regulated due to oxidative stress treatment in the WT and the MacnA mutant (see below). Both harbor predicted IRE motifs on their corresponding transcript sequence. Thus, these data provide a direct *in vivo* evidence for an AcnA-mediated regulation upon oxidative stress.

**Table 1:** Proteins directly regulated by AcnA.

Locus	function	loop sequence of IRE	localization	distance in bp*	fold change	p-value
**WT**						
SSQG_00725	Tellurium resistance protein	CAGUG	5′	64	1.62	0.05
SSQG_01870	Phosphoglycerate kinase	CAGCG	5′	97	1.65	0.05
SSQG_01871	Glyceraldehyde-3-phosphate dehydrogenase	CAGCG	3′	35	1.46	0.02
SSQG_02023	Cell division protein FtsZ	GAGAG	5′	9	1.61	0.03
SSQG_02339	Tellurium resistance protein	GGGAG	3′	100	1.99	0.15
SSQG_03372	Glutathione peroxidase	CAGGG	5′	169	1.99	0.00
SSQG_03670	Phosphate-binding protein PstS	CAGGG	5′	155	0.49	0.07
SSQG_04757	Translation elongation factor Tu	GAGAG	5′	84	0.61	0.01
SSQG_04757	Translation elongation factor Tu	GAGAG	5′	84	0.56	0.04
SSQG_04757	Translation elongation factor Tu	GAGAG	5′	84	1.60	0.07
SSQG_04792	Adenylate kinase	CUGUG	3′	79	2.23	0.02
SSQG_05742	1-deoxy-D-xylulose 5-phosphate reductoisomerase	CAGGG	5′	181	3.05	0.06
SSQG_05905	Conserved hypotetical protein	GAGAG	5′	182	1.54	0.01
**MacnA**						
SSQG_00322	Stress inducible protein	CAGCG	3′	119	0.72	0.03
SSQG_01539	Glycerol operon regulatory protein	GAGAG	5′	90	1.59	0.11
SSQG_02132	Serine protease	CAGGG	3′	33	0.67	0.10
SSQG_02132	Serine protease	CAGGG	3′	44	0.67	0.10
SSQG_02339	Tellurium resistance protein	GGGAG	3′	100	0.41	0.00
SSQG_04757	Translation elongation factor Tu	GAGAG	5′	84	1.38	0.06
SSQG_04757	Translation elongation factor Tu	GAGAG	5′	84	1.29	0.04
SSQG_04757	Translation elongation factor Tu	GAGAG	5′	84	1.55	0.34

* Distance of the IRE-like motifs in base pairs from start or stop codon.

For differentially expressed proteins where no IRE motif was identified on the associated mRNA sequence, the regulation was regarded to be mediated only indirectly by AcnA (Table 2). In such a case AcnA could control the expression of another regulator, which itself directly regulates the respective gene expression. One example of such a regulator could be represented by MarR. The *S. viridochromogenes marR* mRNA sequence has been shown to harbor a predicted IRE element at its 3′ end [5] and thus may be a target of AcnA. MarR is a global regulator and known to be involved in the regulation of a variety of cellular processes including that of stress responses [18]. Thus, this regulator could be a tie point in such a predicted regulatory cascade. However, it also cannot be excluded that the dissimilar expression is based on different physiological preconditions of both strains.

**Table 2:** Proteins indirectly regulated by AcnA.

Locus	function	fold change	p-value
**WT**			
FrEUN1fDRAFT_6253	RNA binding S1 domain protein	1.28	0.04
FrEUN1fDRAFT_6253	RNA binding S1 domain protein	1.83	0.11
ThimaDRAFT_4816	translation elongation factor G	0.59	0.07
HMPREF1013_05577	50S ribosomal protein L5	1.94	0.03
SP187300_0321	50S ribosomal protein L5 (BL6)	3.23	0.10
Niako_6338	tRNA uridine 5-carboxymethylaminomethyl modification enzyme MnmG	1.82	0.17
SCO4729	DNA-directed RNA polymerase subunit alpha	1.70	0.11
SSQG_04836	chaperonin GroL	1.22	0.00
SSQG_04836	chaperonin GroL	1.85	0.01
SSQG_04185	chaperone DnaK	1.52	0.04
SSQG_04185	chaperone DnaK	2.19	0.13
SSQG_05867	protease	1.36	0.13
SSQG_03555	tellurium resistance protein	1.68	0.04
SSQG_03555	tellurium resistance protein	2.10	0.02
SSQG_00870	6-phosphogluconate dehydrogenase NAD-binding protein	1.65	0.02
SSQG_00870	6-phosphogluconate dehydrogenase NAD-binding protein	1.66	0.02
TBCG_03283	isocitrate dehydrogenase [NADP] Icd1	1.66	0.12
SSQG_02359	3-oxoacyl-[acyl-carrier-protein] synthase 2	4.24	0.09
CV_2767	aspartate-semialdehyde dehydrogenase	1.80	0.02
BURMUCGD1_1985	putative cellulose synthase operon protein C	1.54	0.04
FraEuI1c_1109	guanosine pentaphosphate synthetase I/polyribonucleotide nucleotidyltransferase	2.52	0.01
SSQG_03741	phosphoribosylaminoimidazole-succinocarboxamide synthetase	1.61	0.02
RS9917_04255	hypothetical protein RS9917_04255	1.74	0.01
SSQG_05193	conserved hypothetical protein	1.98	0.10
SSQG_02286	conserved hypothetical protein	2.03	0.17
SSQG_04729	conserved hypothetical protein	4.20	0.00
G11MC16DRAFT_3049	conserved hypothetical protein	1.64	0.07
SSQG_03600	conserved hypothetical protein	1.79	0.02
FrEUN1fDRAFT_6253	RNA binding S1 domain protein	0.59	0.07
SSQG_04836	chaperonin GroL	0.61	0.09
BBAL3_51	TldD/PmbA family	0.69	0.03
SSTG_00732	glyceraldehyde-3-phosphate dehydrogenase, type I	0.55	0.08
SCO3096	phosphopyruvate hydratase (enolase)	0.52	0.02
SCO3096	phosphopyruvate hydratase (enolase)	0.51	0.01
CV_2767	aspartate-semialdehyde dehydrogenase	0.61	0.13
SSQG_01989	bifunctional HisA/TrpF protein	0.68	0.03
APM_0032	conjugative relaxase domain protein	0.63	0.09
APM_0032	conjugative relaxase domain protein	0.48	0.02
SSQG_03895	single-strand binding protein	0.31	0.02
SSQG_01951	two-component system response regulator	0.51	0.03
Cbei_3045	methyl-accepting chemotaxis sensory transducer	0.66	0.15
Acid345_0176	radical SAM protein	0.54	0.16
BFAG_02012	cobalamin biosynthesis protein CobD	0.82	0.01
SSQG_03772	uracil phosphoribosyltransferase	0.64	0.10
SSQG_02905	conserved hypothetical protein	0.49	0.00
SSQG_07592	secreted protein	0.43	0.08
SSQG_07014	secreted protein	0.34	0.04
SSQG_07014	secreted protein	0.46	0.15
SSQG_07014	secreted protein	0.73	0.00
**MacnA**			
SSQG_05040	transcription elongation factor GreA	1.37	0.06
SSQG_05128	translation-associated GTPase	1.24	0.06
SSQG_04311	anti-sigma factor	1.51	0.10
SSQG_04185	chaperone DnaK	1.38	0.11
SSQG_05430	methylmalonyl-CoA epimerase	1.90	0.08
SSQG_05406	ATP synthase F1, beta subunit	2.23	0.10
SSQG_01256	forkhead-associated protein	1.40	0.05
SSQG_02356	malonate decarboxylase, epsilon subunit	2.34	0.13
SSQG_03235	4-(cytidine 5′-diphospho)-2-C-methyl-D-erythritol kinase	1.59	0.06
pO86A1_p160	aminoglycoside-3′-O-phosphotransferase	1.45	0.02
SSQG_01691	secreted protein	1.41	0.05
SSQG_02341	conserved hypothetical protein	1.63	0.04
SSQG_02509	glycyl-tRNA synthetase	0.52	0.04
SSQG_05458	thioredoxin	0.53	0.07
SSQG_01736	3-oxoacyl-[acyl-carrier-protein] reductase	0.69	0.07
SSQG_01640	cell division protein SepF1	4.97	0.20
SSQG_02020	cell division protein SepF2	0.79	0.01
HMPREF0873_01794	ABC transporter, ATP-binding family protein	0.51	0.07
SSQG_06852	monomeric isocitrate dehydrogenase	0.44	0.16
SSQG_05319	dehydrogenase	0.75	0.03

### Aconitase affects expression of proteins involved in cellular protection against oxidative damage

Three proteins (SSQG_03555, SSQG_00725, and SSQG_02339) annotated as putative tellurium resistance proteins, were found to be upregulated in the WT strain under oxidative stress conditions (Table S1). Tellurium resistance proteins represent a family of bacterial stress proteins that mediate resistance to tellurite (TeO_3_^−2^) and other xenobiotic toxic compounds, pore-forming colicins and several bacteriophages [19]. Tellurite acts as a strong oxidizing agent and its reactivity leads to the generation of reactive oxygen species (ROS) in the cytoplasm [20]. For the tellurium resistance protein SCO2368 from *S. coelicolor*, which shares 92% amino acid sequence identity to SSQG_02339, it has been shown that the level of protein increased during incubation of the bacterial cells with plant material. In these analyses a simultaneous increase of superoxide dismutase was observed. Therefore it was suggested that plant material provides some kind of oxidative stress [21], [22]. A similar concomitant increase of tellurium resistance protein and superoxide dismutase was observed for *Frankia* cells incubated with root exudates of *Alnus glutinosa* [23]. Moreover, the induction of tellurium resistance due to oxidative stress (also due to MV supplementation) has been shown for *Rhodobacter capsulatus* [24]. Interestingly, we found putative IRE sequences in front of the genes encoding two out of the three identified tellurium resistance proteins (SSQG_02339, SSQG_00725), suggesting that their expression might be directly controlled by AcnA (Table 1). In particular, protein SSQG_02339 with the predicted IRE motif was found to be upregulated in the WT strain but downregulated in the MacnA. This result indicates that AcnA positively regulates the expression of SSQG_02339 under oxidative stress conditions. A congeneric upregulation of superoxide dismutase was not observed in *S. viridochromogenes*, which may be associated with the type of oxidative stress supply as observed before [25], [26]. Furthermore, as no IRE motif was identified on the superoxide dismutase mRNA sequence, the expression of this protein is not expected to be directly regulated by AcnA.

The putative stress inducible protein SSQG_00322 belongs to the universal stress protein family Usp and was downregulated in MacnA due to oxidative stress treatment. In *E. coli* the production of Usp proteins is stimulated by a broad range of conditions including starvation for carbon, antibiotic treatment, heat or oxidative stress, etc. [27]. Furthermore, Usp expression was increased in hypoxic cells of *Mycobacterium tuberculosis* and *Mycobacterium smegmatis* [28], [29] and also was important for *Pseudomonas aeruginosa* survival under anaerobic energy stress [30]. However, the exact biological function of these proteins is not known so far. Interestingly, at the 3′ end of the Usp encoding gene sequence in *S. viridochromogenes* an IRE motif was identified, which hints to a post-transcriptional regulation by AcnA. Thus, the observed downregulation of SSQG_00322 in the MacnA mutant might be due to the lack of an AcnA-mediated regulation.

An IRE motif was also identified upstream of a gene, encoding a putative glutathione peroxidase (SSQG_03372). The respective protein was significantly upregulated in the WT strain upon MV treatment. Glutathione peroxidases are among the critical enzymes that are needed to maintain the cytoplasmic redox potential and to protect organisms from oxidative damage [31].

The protein SSQG_05458 that codes for a putative thioredoxin was found to be significantly downregulated in MacnA in response to oxidative stress conditions. Thioredoxins are small redox proteins, which function as antioxidants by directly reducing hydrogen peroxide and also disulfide bonds formed by ROS [32]. The role of thioredoxins in oxidative stress response has been described for several bacteria, such as *E. coli*, *Mycobacterium leprae*, *Helicobacter pylori*, or *S. coelicolor* [33]–[36]. Normally, the level of thioredoxin is expected to increase after oxidative stress treatment. Interestingly, in our MacnA mutant the level of SSQG_05458 strongly decreased under such conditions.

A putative serine protease (SSQG_02132) was found to be downregulated upon MV treatment in MacnA and the respective mRNA sequence turned out to possess two IRE motifs (Table 1). Both are located in close proximity of the translational stop codon. The presence of two IRE motifs located so closely to each other is rather seldom observed. Perhaps two IRE motifs at the 3′ end provide stronger aconitase binding and consequently a better transcript stabilization and protection. Serine proteases have already been shown to play a role in protection against oxidative damage [37], [38]. However, the mechanism of this protection is not fully understood. For the HtrA family of serine proteases of Gram-negative bacteria it was assumed that they degrade an excess of misfolded or denaturated proteins [39].

In conclusion, many of the identified proteins turned out to have a function in sensing, preventing or overcoming oxidative stress. This gives us a general view of which proteins are crucial for *S. viridochromogenes*' adaptation to oxidative stress conditions and also a notion about the cellular preconditions that may underlay the high oxygen stress sensitivity of the *S. viridochromogenes* aconitase mutant. For example, the downregulation of proteins such as thioredoxin (SSQG_05458), stress inducible protein (SSQG_00322), and tellurium resistance protein (SSQG_02339) may significantly contribute to the impaired defense of MacnA against free radicals.

### Aconitase controls EF-Tu expression upon oxidative stress

A set of differentially regulated, putative transcription and translation proteins, were identified in both strains. In the WT, the majority of translation-related proteins, such as 50S ribosomal protein L5 (HMPREF1013_05577, SP187300_0321), RNA binding S1 domain protein (FrEUN1fDRAFT_6253), tRNA uridine 5-carboxymethylaminomethyl modification enzyme MnmG (Niako_6338), translation elongation factor G (ThimaDRAFT_4816), as well as the alpha subunit of transcription enzyme DNA-directed RNA polymerase (SCO4729) were found to be upregulated after oxidative stress induction. Among the translation and transcription proteins upregulated in MacnA were the translation-associated GTPase (SSQG_05128), the transcription elongation factor GreA (SSQG_05040), the anti-sigma factor (SSQG_04311), and the translation elongation factor Tu (EF-Tu) (SSQG_04757). Interestingly, almost all isoforms of the putative EF-Tu protein (SSQG_04757) were significantly downregulated in the WT strain under the same stress conditions (Table S1). The downregulation of this protein in the WT and its upregulation in MacnA suggests that AcnA negatively influences the SSQG_04757 expression. This regulation may be governed directly by AcnA, since an IRE motif was identified at the 5′ end of the respective mRNA sequence (Table 1). This is another direct *in vivo* evidence for an AcnA-mediated regulation as observed vice versa for the expression of the tellurium resistance protein SSQG_02339.

EF-Tu is a highly abundant bacterial protein (5–10% of the total proteins) [40] that promotes the GTP-dependent binding of the aminoacyl-tRNA to the A-site of the ribosome during protein biosynthesis and contributes to translational accuracy. It was also one of the most abundant proteins in *S. viridochromogenes* WT and MacnA. Because of the central role of EF-Tu, its downregulation in the WT strain may lead to a significant reduction in protein synthesis. Perhaps such a downregulation has a function in reprograming cell metabolism to divert resources away from extensive protein production under stress conditions towards amino acid synthesis in order to promote survival, which could assist the process of stringent response (see below). Besides the well-established function of EF-Tu in protein synthesis, it is also involved in other cellular processes, including cell shape maintenance as shown for *B. subtilis* [41] or protection of proteins during heat stress in *E. coli* [42]. Contrary to our results, in proteome analysis of *E. coli*, EF-Tu reached its maximum expression as a result of the exposure of the strain to different environmental stress conditions [43]. Here it was suggested that this overexpression might be related with the chaperon function of EF-Tu [42], [43]. In *E. coli* EF-Tu interacts with unfolded and denaturated proteins. For example, it promotes the correct folding of citrate synthase preventing its aggregation under heat shock conditions [42]. Even if it is hard to drive a firm conclusion, one can say that stress conditions have a significant influence on the cellular EF-Tu content.

### AcnA-mediated regulation may contribute to carbon flux redistribution under oxidative stress

SSQG_00870 is a putative 6-phosphogluconate dehydrogenase that was significantly upregulated in the WT strain upon oxidative stress treatment. 6-phosphogluconate dehydrogenase is a key enzyme in the pentose phosphate pathway, where it catalyzes the reaction from 6-phosphogluconate to ribulose-5-phosphate. This reaction is especially important for NADPH generation. NADPH provides reducing equivalents for the biosynthetic reactions and also for redox reactions involved in protection processes against ROS, such as the reduction of oxidized forms of glutathione and thioredoxin [44]. Thus, NADPH is a pivotal component of the oxidative stress defense. In yeast, a 6-phosphogluconate dehydrogenase mutant showed increased sensitivity to hydrogen peroxide, revealing the importance of this enzyme in protection against oxidative stress [45]. As it has been shown that redox cycling of MV leads to a destructive oxidation of NADPH [46], the upregulation of SSQG_00870 in *S. viridochromogenes* may meet the increased demand for reducing equivalents under oxidative stress conditions. However, this regulation might be only indirectly mediated by AcnA as no IRE motif was identified on the respective 6-phosphogluconate dehydrogenase gene sequence.

Also enzymes involved in glycolysis were identified in the WT to be differentially expressed during the period of oxidative stress. The phosphopyruvate hydratase (SCO3096) was downregulated, whereas phosphoglycerate kinase (SSQG_01870) was upregulated. On the corresponding gene sequence of SSQG_01870, an IRE motif was identified, which suggests an AcnA-mediated regulation of the respective mRNA (Table 1). For *Caenorhabditis elegans* it has been shown that the oxidative inhibitions of other glycolytic enzymes, such as glyceraldehyde 3-phosphate dehydrogenase (GADPH) or phosphate isomerase (TPI), leads to an increased resistance of the strain to oxidative stress [47]. This is because of the redirection of the carbohydrate flux from glycolysis to the pentose phosphate pathway, which results in the generation of NADPH and in this way supports the oxidative stress defense [47]. Perhaps a similar redirection of the carbohydrate flux as a contribution to oxidative stress defense exists in *S. viridochromogenes*. In our analysis the putative glyceraldehyde-3-phosphate dehydrogenase (GAPDH) (SSQG_01871, SSTG_00732) was also identified in the WT, however, since the protein was once up- and once downregulated, the meaning of its regulation is difficult to assess.

An IRE sequence was also identified upstream of the gene encoding the putative glycerol operon regulatory protein 302550310, which was found to be upregulated after MV treatment in MacnA. SSQG_01539 shows 94% identity on amino sequence level to the glycerol operon regulatory protein GylR of *S. coelicolor*, which is a repressor of the *gyl*CABX operon that is involved in glycerol catabolism [48]. The upregulation of SSQG_01539 in the aconitase mutant suggests that AcnA may negatively influence the SSQG_01539 expression. Glycerol is an important metabolite in cellular response to oxidative stress as for example it functions as an efficient free radicals scavenger and is crucial for the regulation of the redox processes [49]. Glycerol synthesis seems to be required for tolerance to MV in yeast [50]. Studies in *Entamoeba histolytica* and *Saccharomyces cerevisiae* suggest that the oxidative stress results in the inhibition of glycolysis leading to the redirection of the carbohydrate flux towards the regeneration of NADPH and glycerol production [49], [51]. Consequently, in our analysis, the overexpression of the putative repressor SSQG_01539 of the glycerol catabolism pathway in MacnA can be regarded as an adaptation to the oxidative stress conditions, which probably leads to an accumulation of glycerol and regeneration of reducing equivalents.

A putative NADP-dependent isocitrate dehydrogenase (IDH-NADP) TBCG_03283 was found to be upregulated in response to oxidative stress in the WT strain. This protein was identified as homologue of *M. tuberculosis*. IDH-NADP is one of the key enzymes of the TCA cycle and catalyzes the reaction from isocitrate to α-ketoglutarate by generating one molecule of NADPH. The upregulation of TBCG_03283 is in agreement to what has been observed in *Pseudomonas fluorescens*, where the activity and expression of IDH-NADP were increased after exposure to an oxidative environment [52]. The overexpression of IDH-NADP leads to an increased NADPH level and in this way contributes to the metabolic adaptation to oxidative stress conditions [52]. In contrast, a downregulation of the putative IDH-NADP SSQG_06852 was observed in MacnA. Thus this downregulation may lead to a cellular NADP^+^\NADPH imbalance, which could contribute to the oxidative stress sensitivity of MacnA. As the IDH-NADP was differentially expressed in the WT and in the aconitase mutant strain, the expression of this enzyme seems to be regulated by AcnA. However, as no IRE motif was identified on the respective IDH-NADP gene sequence, this regulation may be only indirect.

Altogether, our observations show that carbon metabolism pathways of *S. viridochromogenes*, such as pentose phosphate pathway, glycolysis and TCA cycle are significantly influenced by oxidative stress and that these changes may cause metabolic flux redistributions towards the generation of NADPH as protective response.

### Aconitase regulates expression of proteins involved in cell growth and morphological differentiation

The cell division protein FtsZ (SSQG_02023) was significantly upregulated in the WT strain after MV application. By gel shifts assays it has been shown before that the *ftsZ* IRE motif is a target for AcnA binding, which strongly suggests an AcnA-mediated regulation of FtsZ expression [5]. The fact that SSQG_02023 was not identified in MacnA among the proteins with significant change in expression may be because of the missing AcnA-mediated regulation of this protein.

Two other putative cell division proteins associated with FtsZ, SepF1 (SSQG_01640) and SepF2 (SSQG_02020), were downregulated under oxidative stress conditions in MacnA. Moreover, a protein identified as secreted protein (SSQG_01691) was upregulated in MacnA, which is similar to the sporulation control protein Spo0M of *B. subtilis* [53].

Taking together, our proteomic results suggest that *S. viridochromogenes* undergoes some regulation of proteins involved in cell division and sporulation processes upon oxidative stress. The regulation of these proteins may be either mediated directly by AcnA as described for FtsZ or indirectly, such as in the case of the SepF1, SepF2 or Spo0M homologous proteins.

### AcnA controls expression of proteins involved in phosphate uptake and phosphate metabolism

The putative PstS protein (SSQG_03670) was downregulated in the WT strain due to oxidative stress. Upstream of the *pstS* gene an IRE motif was identified, which suggests an AcnA-mediated regulation of the respective protein expression (Table 1). Pst proteins generally function as high-affinity-phosphate binding proteins [54], [55]. For *Lactococcus lactis* it has been shown that inactivation of the *pst* genes, including *pstS*, results in higher resistance to oxidizing agents such as tellurite and H_2_O_2_ [56]–[58]. It was speculated that the inactivation of the major phosphate uptake pathway mimics phosphate starvation and induces a number of stress responses that cause cross-resistance to different stressors [56], [57]. For example, in *Salmonella typhimurium* different proteins were overproduced upon phosphate starvation [59]. Induction of these proteins contributes to cell survival under various stress conditions [57], [60]. The downregulation of PstS upon oxidative stress in the *S. viridochromogenes* wild-type most likely also leads to phosphate starvation, which in turn may cause the elevated expression of stress response genes and thus a better cellular defense under this unfavorable condition.

A number of environmental stress conditions, including phosphate starvation can be signaled in cells by the alarmone guanosine pentaphosphate (p)ppGpp, which mediates the so-called stringent response [61], [62]. Stringent response allows bacteria to quickly reprogram transcription in response to nutrient limitation or stress signals [63]. Via ppGpp several metabolic pathways are inhibited, such as RNA synthesis or protein synthesis, whereas others like those that are needed for the exploitation of nutrient sources, protein degradation or the synthesis of proteins for stress combat are activated. In *E. coli* the ppGpp production was observed to be induced as a response to the exposure to MV and H_2_O_2_ [64], [65]. In our analysis a protein identified as guanosine pentaphosphate synthetase I/polyribonucleotide nucleotidyltransferase (FraEuI1c_1109), which is suggested to be responsible for (p)ppGpp biosynthesis, was upregulated in the WT strain upon MV treatment. This indicates that FraEuI1c_1109 is involved in adaptation of *S. viridochromogenes* to oxidative stress.

### Proteins of fatty acids metabolism respond to oxidative stress conditions via indirect AcnA-mediated regulation

Three proteins were identified as putative enzymes involved in fatty acid metabolism: 3-oxoacyl-[acyl-carrier-protein] synthase (SSQG_02359), which was upregulated in the WT strain; methylmalonyl-CoA epimerase (SSQG_05430) upregulated in MacnA; and 3-oxoacyl-[acyl-carrier-protein] reductase (SSQG_01736) that was downregulated in MacnA due to oxidative stress treatment. Fatty acid metabolism influences the cellular membrane lipid composition. The saturation or unsaturation of membrane lipids is one of the factors determining the chance of survival of prokaryotic and eukaryotic cells under environmental stress conditions [66], [67]. This has been shown for *S. coelicolor*, *S. cerevisiae*, *B. subtilis*, and *Bacillus megaterium*, which were more resistant to H_2_O_2_ or high temperature stress the higher the content of saturated fatty acid in their membrane lipids was [68]–[69]. For *S. viridochromogenes* it is known that fatty acids composition of their membrane lipids has a crucial effect on membrane rigidity and permeability [70]. Our data of the changed expression of proteins contributing to fatty acids metabolism also may reflect the importance of membrane lipid composition in oxidative stress response of *S. viridochromogenes*. As the corresponding mRNA sequences of the identified proteins were not found to possess any IRE element, their expression may only be indirectly mediated by AcnA.

### The chaperones DnaK and GroL are a part of general oxidative stress response in *S. viridochromogenes*

In our analysis, DnaK (SSQG_04185) and GroL (SSQG_04836) were among the most abundant proteins identified in the WT strain after oxidative stress treatment. DnaK and GroL are stress- inducible chaperones that function in preventing protein misfolding and aggregation of potentially toxic proteins [71]. Our result correspond with the proteome data from *Deinococcus geothermalis*, where DnaK, GroEL and GroES were also detected as the predominant proteins in that strain, which itself is known for its capacity to overcome extremely harsh conditions including oxidative stress [72]. In our analysis DnaK and GroL were identified in both strains as multiple spots, which suggest post-transcriptional modifications of these proteins. All these spots, except one isoform of GroL, were upregulated in both, the WT and the MacnA strain, obviously as part of a general response of *S. viridochromogenes* to oxidative stress. Thus, as the upregulation was observed in both strains, this is suggested to happen independently from any regulation by AcnA.

Summing up, our proteome data together with the *in silico* analyses reveal evidences for a direct AcnA-mediated regulation upon oxidative stress and also give insight into the oxidative stress adaptation of *S. viridochromogenes*. In prospective experiments the regulatory binding capability of AcnA to the newly identified target mRNAs will be analyzed by *in vitro* shift studies and mutational analyses.

## Supporting Information

Figure S1 **Growth curve of** ***S. viridochromogenes*** **wild-type (A) and MacnA (B).** Time point of methyl viologen (MV) supply is indicated as black arrow.
(TIF)

Figure S2 **2D gel image of the methyl viologen-treated** ***S. viridochromogenes*** **wild-type (A) and untreated wild-type (B).** 2D gel image of the methyl viologen-treated *S. viridochromogenes* MacnA (**C**) and untreated MacnA (**D**). Significantly changed spots are outlined in green. They showed a statistically significant variation of spot volume and minimal fold variation of 1.1. These spots (73 for WT and 36 for MacnA) were excised for subsequent protein identification by mass spectrometry. White arrows indicate the spots that were identified in both, WT and MacnA strain (see Table 1 for code assignment).
(TIF)

Table S1 **Proteins differentially expressed in** ***S. viridochromogenes*** **WT and MacnA due to oxidative stress treatment identified by MALDI TOF/TOF**
(DOCX)
