# Supplementary figures and images for: Proteomic Approach to Reveal the Regulatory Function of Aconitase AcnA in Oxidative Stress Response in the Antibiotic Producer Streptomyces viridochromogenes Tü494
Source: PLoS One. 2014 Feb 3;9(2):e87905. doi: 10.1371/journal.pone.0087905 (PMC3912134; doi:10.1371/journal.pone.0087905)

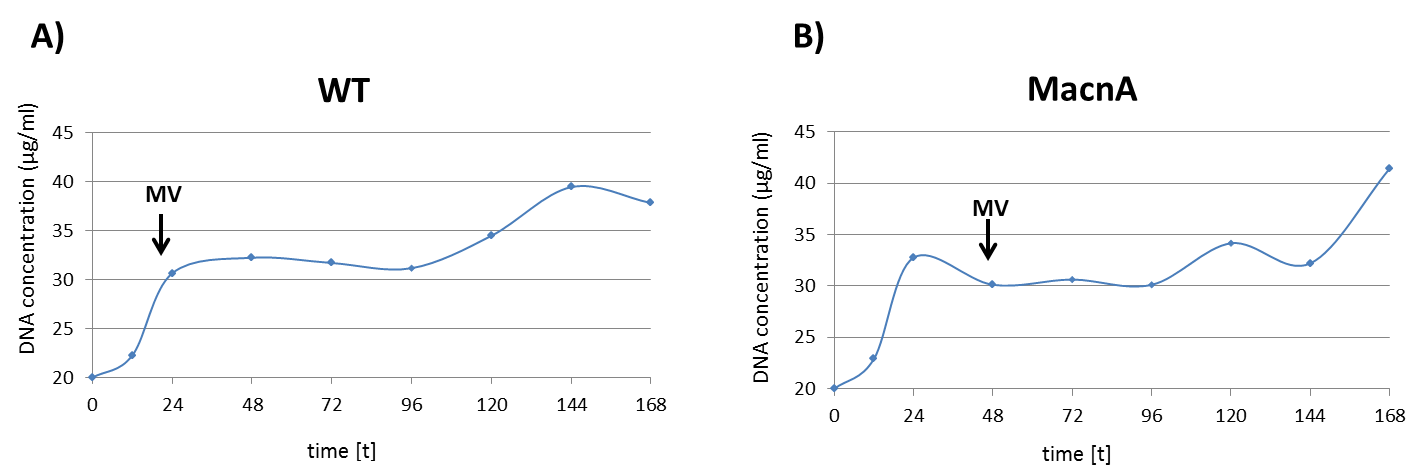

Supplement: Figure S1 — Growth curve of S. viridochromogenes wild-type (A) and MacnA (B). Time point of methyl viologen (MV) supply is indicated as black arrow. (TIF) [file pone.0087905.s001.tif]

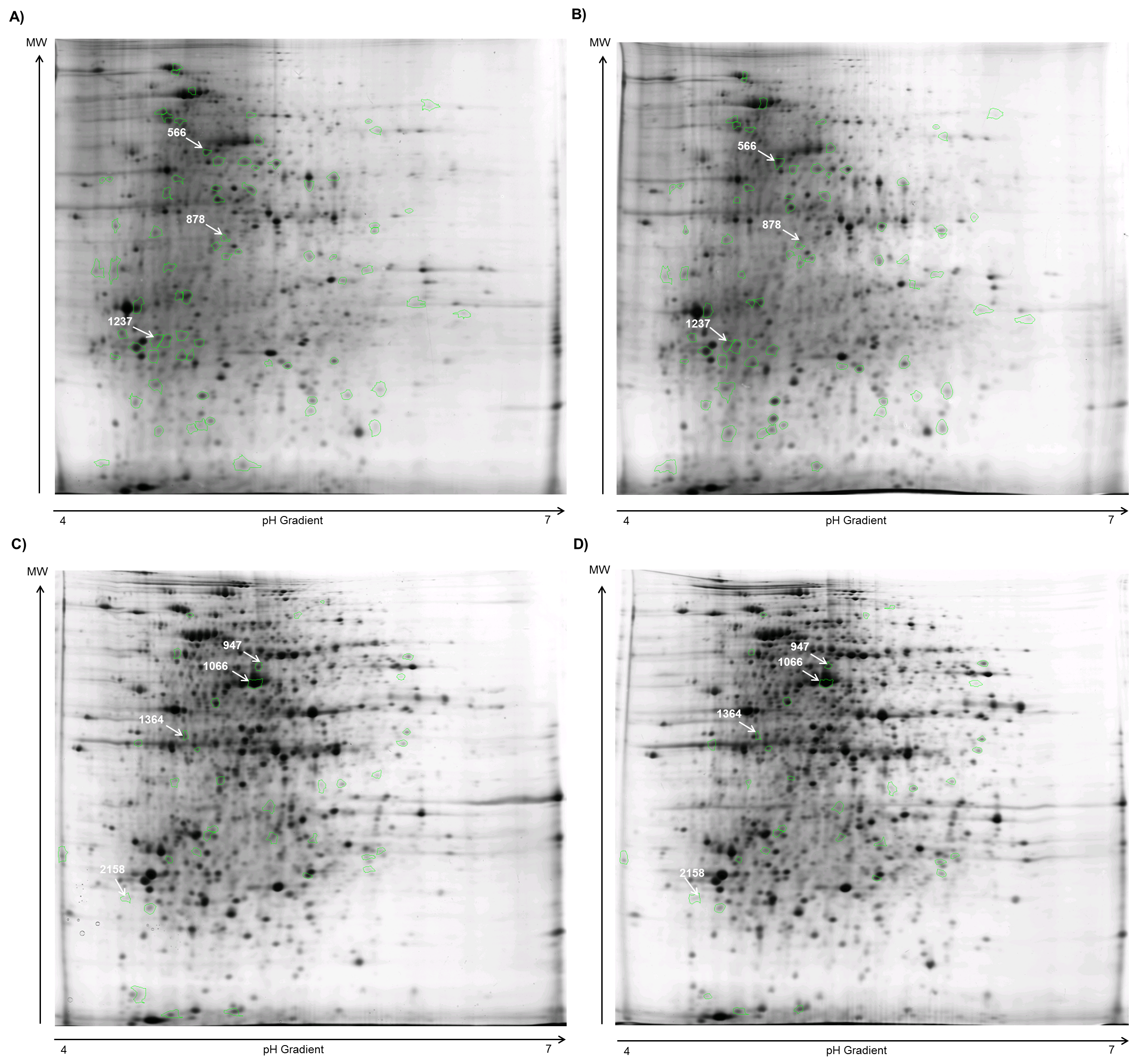

Supplement: Figure S2 — 2D gel image of the methyl viologen-treated S. viridochromogenes wild-type (A) and untreated wild-type (B). 2D gel image of the methyl viologen-treated S. viridochromogenes MacnA (C) and untreated MacnA (D). Significantly changed spots are outlined in green. They showed a statistically significant variation of spot volume and minimal fold variation of 1.1. These spots (73 for WT and 36 for MacnA) were excised for subsequent protein identification by mass spectrometry. White arrows indicate the spots that were identified in both, WT and MacnA strain (see Table 1 for code assignment). (TIF) [file pone.0087905.s002.tif]
