# Supplementary material for: Proteomic Approach to Reveal the Regulatory Function of Aconitase AcnA in Oxidative Stress Response in the Antibiotic Producer Streptomyces viridochromogenes Tü494
Source: PLoS One. 2014 Feb 3;9(2):e87905. doi: 10.1371/journal.pone.0087905 (PMC3912134; doi:10.1371/journal.pone.0087905)
Supplement: Table S1 — Proteins differentially expressed in S. viridochromogenes WT and MacnA due to oxidative stress treatment identified by MALDI TOF/TOF (DOCX) [file pone.0087905.s003.docx]

| **Table S1 Proteins differentially expressed in *S. viridochromogenes* WT and MacnA due to oxidative stress treatment identified by MALDI TOF/TOF** | | | | | | | | | | | | | | | | | | | |
| --- | --- | --- | --- | --- | --- | --- | --- | --- | --- | --- | --- | --- | --- | --- | --- | --- | --- | --- | --- |
| Spo no. | Locus | Gene | | Protein name | Fold change | | p-value | Presence of IRE | Ratio (relative spot volume) | | Theoretical/experiment.mass (kDa) | Theoretical/experiment.pI | Proten score C.I% | Total ion score | | Peptides matched | | Microorganism | Function/Pathway |
| **WT, upregulated proteins** | | | |  |  | |  |  |  | |  |  |  |  | |  | |  |  |
| **Protein synthesis and protein turnover** | | | | | | | | | | | | | | | | | | | |
| 661 | FrEUN1fDRAFT_6253 |  | | RNA binding S1 domain protein | 1.28 | | 0.04 | **-** | 1.11 | 82.7/66 | | 5.28/4.9 | 97.27 | 31 | 12 | | [*Frankia sp.* EUN1f] | | Protein biosynthesis/ Translation |
| 409 | FrEUN1fDRAFT_6253 |  | | RNA binding S1 domain protein | 1.83 | | 0.11 | **-** | 1.31 | 82.7/66 | | 5.28/6.0 | 98.78 | 43 | 10 | | [*Frankia sp.* EUN1f] | | Protein biosynthesis/ Translation |
| 520 | SSQG_04757 |  | | translation elongation factor Tu | 1.60 | | 0.07 | **+** | 1.34 | 43. 9/48 | | 5.0/5.3 | 100 | 692 | 15 | | [*Streptomyces viridochromogenes* DSM 40736] | | Protein biosynthesis/ Translation |
| 1421 | HMPREF1013_05577 | *rp1E* | | 50S ribosomal protein L5 | 1.94 | | 0.03 | **-** | 1.25 | 20.2/25 | | 9.77/5.8 | 97.4 | 46 | 7 | | [*Bacillus sp.* 2_A_57_CT2] | | Protein biosynthesis/ Translation |
| 1290 | SP187300_0321 | *rp1E* | | 50S ribosomal protein L5 (BL6) | 3.23 | | 0.10 | **-** | 1.77 | 19.79/25 | | 9.04/5.0 | 95.68 | 42 | 6 | | [*Streptococcus pneumoniae*  CDC1873-00] | | Protein biosynthesis/ Translation |
| 970 | Niako_6338 |  | | tRNA uridine 5-carboxymethylaminomethyl modyfication enzyme MnmG | 1.82 | | 0.17 | **-** | 1.11 | 69.5/50 | | 6.14/4.5 | 95.37 |  | 15 | | [*Niastella koreensis* GR20-10] | | Protein biosynthesis/ Translation-tRNA processing |
| 697 | SCO4729 | *rpoA* | | DNA-directed RNA polymerase subunit alpha | 1.70 | | 0.11 | **-** | 1.17 | 36.73/40 | | 4.66/5.1 | 100 | 216 | 15 | | [*Streptomyces coelicolor* A3(2)] | | Protein biosynthesis/ Transcription |
| 423 | SSQG_04836 | *groEL* | | chaperonin GroL | 1.22 | | 0.00 | **-** | 1.16 | 56.85/60 | | 4.86/4.8 | 97.78 | 20 | 11 | | [*Streptomyces viridochromogenes* DSM 40736] | | Protein folding and stabilisation |
| 444 | SSQG_04836 | *groEL* | | chaperonin GroL | 1.85 | | 0.01 | **-** | 1.51 | 56.85/60 | | 4.86/4,8 | 100 | 178 | 15 | | [*Streptomyces viridochromogenes* DSM 40736] | | Protein folding and stabilisation |
| 265 | SSQG_04185 | *dnaK* | | chaperone DnaK | 1.52 | | 0.04 | **-** | 1.18 | 66.2/75 | | 4.76/4.8 | 100 | 249 | 10 | | [*Streptomyces viridochromogenes* DSM 40736] | | Protein folding and stabilisation |
| 282 | SSQG_04185 | *dnaK* | | chaperone DnaK | 2.19 | | 0.13 | **-** | 1.32 | 66.19/66 | | 4.76/4.8 | 100 | 237 | 9 | | [*Streptomyces viridochromogenes* DSM 40736] | | Protein folding and stabilisation |
| 467 | SSQG_05867 |  | | protease | 1.36 | | 0.13 | **-** | 1.12 | 48.8/60 | | 4.87/4.9 | 100 | 242 | 13 | | [*Streptomyces viridochromogenes* DSM 40736] | | Protein degradation |
| **Stress proteins** | | | | | | | | | | | | | | | | | | | |
| 1271 | SSQG_03555 | | *terD, terZ* | tellurium resistance protein | 1.68 | | 0.04 | **-** | 1.22 | 20.27/35 | | 4.53/4.7 | 100 | 854 | 9 | | [*Streptomyces viridochromogenes* DSM 40736] | | Tellurium resistance/ Stress response |
| 1299 | SSQG_03555 | | *terD, terZ* | tellurium resistance protein | 2.10 | | 0.02 | **-** | 1.72 | 20.27/35 | | 4.53/4.6 | 100 | 533 | 9 | | [*Streptomyces viridochromogenes* DSM 40736] | | Tellurium resistance/ Stress response |
| 1237 | SSQG_02339 | | *terD, terZ* | tellurium resistance protein | 1.99 | | 0.15 | **+** | 1.60 | 20.38/25 | | 4.54/4.8 | 100 | 395 | 10 | | [*Streptomyces viridochromogenes* DSM 40736] | | Tellurium resistance/ Stress response |
| 1293 | SSQG_00725 | | *terD, terZ* | tellurium resistance protein | 1.62 | | 0.05 | **+** | 1.19 | 20.19/25 | | 4.55/4.8 | 100 | 328 | 8 | | [*Streptomyces viridochromogenes* DSM 40736] | | Tellurium resistance/ Stress response |
| 1406 | SSQG_03372 | |  | glutathione peroxidase | 1.99 | | 0.00 | **+** | 1.55 | 17.99/22 | | 4.53/4.6 | 99.72 | 79 | 3 | | [*Streptomyces viridochromogenes* DSM 40736] | | Oxidative stress response |
| **Carbon metabolism** | | | | | | | | | | | | | | | | | | | |
| 1450 | SSQG_01871 | |  | glyceraldehyde-3-phosphate dehydrogenase | 1.46 | | 0.02 | + | 1.20 | 36.34/25 | | 5.27/5.1 | 100 | 123 | 4 | | [*Streptomyces viridochromogenes* DSM 40736] | | Glycolysis |
| 691 | SSQG_01870 | |  | phosphoglycerate kinase | 1.65 | | 0.05 | **+** | 1.25 | 41.8/40 | | 5.1/5.1 | 100 | 384 | 10 | | [*Streptomyces viridochromogenes* DSM 40736] | | Glycolysis |
| 1116 | SSQG_00870 | | *mmsB* | 6-phosphogluconate dehydrogenase NAD-binding protein | 1.65 | | 0.02 | **-** | 1.34 | 30.77/31 | | 4.52/4.6 | 100 | 600 | 11 | | [*Streptomyces viridochromogenes* DSM 40736] | | Pentose phosphate pathway |
| 1208 | SSQG_00870 | | *mmsB* | 6-phosphogluconate dehydrogenase NAD-binding protein | 1.66 | | 0.02 | **-** | 1.38 | 30.77/18 | | 4.52/4,5 | 100 | 344 | 9 | | [*Streptomyces viridochromogenes* DSM 40736] | | Pentose phosphate pathway |
| 1011 | TBCG_03283 | | *icd1* | isocitrate dehydrogenase [NADP] Icd1 | 1.66 | | 0.12 | **-** | 1.52 | 46.17/35 | | 5.13/5.8 | 93.15 |  | 10 | | [*Mycobacterium tuberculosis C*] | | TCA cycle |
| **Energy metabolism** | | | | | | | | | | | | | | | | | | | |
| 1072 | SSQG_04792 | | *adk* | adenylate kinase | 2.23 | | 0.02 | **+** | 1.57 | 24.39/30 | | 4.78/5.0 | 100 | 243 | 7 | | [*Streptomyces viridochromogenes* DSM 40736] | | ATP production/ consumption |
| **Fatty acids metabolism** | | | | | | | | | | | | | | | | | | | |
| 595 | SSQG_02359 | | *fabF* | 3-oxoacyl-[acyl-carrier-protein] synthase 2 | 4.24 | | 0.09 | **-** | 1.50 | 43.65/50 | | 5.19/5.4 | 100 | 589 | 11 | | [*Streptomyces viridochromogenes* DSM 40736] | | Fatty acids biosynthesis |
| **Others** | | | | | | | | | | | | | | | | | | | |
| 1110 | CV_2767 | | *dhC* | aspartate-semialdehyde dehydrogenase | 1.80 | | 0.02 | **-** | 1.52 | 35.9/25 | | 4.92/6.0 | 96.57 | 54 | 6 | | [*Chromobacterium violaceum* ATCC 12472] | | Amino acids biosynthesis |
| 582 | SSQG_05742 | |  | 1-deoxy-D-xylulose 5-phosphate  reductoisomerase | 3.05 | | 0.06 | **+** | 1.31 | 43.92/43 | | 5.03/5.2 | 100 | 363 | 16 | | [*Streptomyces viridochromogenes* DSM 40736] | | Non-mevalonate pathway of terpenoid synthesis |
| 707 | BURMUCGD1_1985 | |  | putative cellulose synthase operon protein C | 1.54 | | 0.04 | **-** | 1.27 | 14.12/40 | | 8.88/5.0 | 99.96 |  | 22 | | [*Burkholderia multivorans* CGD1] | | Cellulose biosynthesis |
| 912 | FraEuI1c_1109 | |  | guanosine pentaphosphate synthetase I/polyribonucleotide nucleotidyltransferase | 2.52 | | 0.01 | **-** | 1.89 | 77.53/38 | | 5.03/5.2 | 100 | 114 | 9 | | [*Frankia sp*.EuI1c] | | Signal transduction/amino acids starvation signalling |
| 866 | SSQG_03741 | |  | phosphoribosylaminoimidazole-succinocarboxamide synt. | 1.61 | | 0.02 | **-** | 1.41 | 33.19/35 | | 4.63/4.8 | 100 | 156 | 10 | | [*Streptomyces viridochromogenes* DSM 40736] | | Purine metabolism |
| 822 | SSQG_02023 | | *ftsZ* | cell division protein FtsZ | 1.61 | | 0.03 | **+** | 1.17 | 40.79/40 | | 4.42/4.5 | 100 | 391 | 12 | | [*Streptomyces viridochromogenes* DSM 40736] | | Cellular growth |
| 978 | RS9917_04255 | |  | hypothetical protein RS9917_04255 | 1.74 | | 0.01 | **-** | 1.42 | 23.24/35 | | 10.89/6.0 | 98.35 |  | 9 | | [*Synechococcus sp.* RS9917] | | Putative transcriptional regulator |
| 1227 | SSQG_05193 | |  | conserved hypothetical protein | 1.98 | | 0.10 | **-** | 1.24 | 20.69/25 | | 4.77/4.8 | 100 | 112 | 9 | | [*Streptomyces viridochromogenes* DSM 40736] | | Putative supressor of fused protein (SUFU) |
| 1520 | SSQG_04729 | |  | conserved hypothetical protein | 4.20 | | 0.00 | **-** | 3.37 | 16.28/16 | | 5.05/5.2 | 100 | 206 | 6 | | [*Streptomyces viridochromogenes* DSM 40736] | | Putative enzyme from polyester biosynthetic pathway |
| 965 | SSQG_05905 | |  | conserved hypothetical protein | 1.54 | | 0.01 | **+** | 1.37 | 26.5/30 | | 4.49/4.5 | 100 | 232 | 11 | | [*Streptomyces viridochromogenes* DSM 40736] | | Unknown |
| 1402 | SSQG_03600 | |  | conserved hypothetical protein | 1.79 | | 0.02 | **-** | 1.29 | 27.08/22 | | 9.36/5.7 | 99.99 | 76 | 7 | | [*Streptomyces viridochromogenes* DSM 40736] | | Unknown |
| **WT, downregulated proteins** | | | |  |  | |  |  |  |  | |  |  |  |  | |  | |  |
| **Protein synthesis and protein turnover** | | | | | | | | | | | | | | | | | | | |
| 943 | FrEUN1fDRAFT_6253 | |  | RNA binding S1 domain protein | 0.59 | | 0.07 | **-** | 1.12 | 82.70/40 | | 5.28/5.7 | 96.23 | 39 | 10 | | [*Frankia sp.* EUN1f] | | Protein biosynthesis/ Translation |
| 878 | SSQG_04757 | |  | translation elongation factor Tu | 0.61 | | 0.01 | **+** | 1.32 | 43.79/35 | | 5.0/5.2 | 100 | 185 | 7 | | [*Streptomyces viridochromogenes* DSM 40736] | | Protein biosynthesis/ Translation |
| 566 | SSQG_04757 | |  | translation elongation factor Tu | 0.56 | | 0.04 | **+** | 1.24 | 43.79/46 | | 5.0/5.0 | 100 | 585 | 12 | | [*Streptomyces viridochromogenes* DSM 40736] | | Protein biosynthesis/ Translation |
| 946 | ThimaDRAFT_4816 | |  | translation elongation factor G | 0.59 | | 0.07 | **-** | 1.12 | 77.45/45 | | 5.2/5.1 | 99.68 |  | 19 | | [*Thiocapsa marina 5811*] | | Protein biosynthesis/ Translation |
| 339 | SSQG_04836 | | *groEL* | chaperonin GroL | 0.61 | | 0.09 | **-** | 1.14 | 56.85/60 | | 4.86/4.9 | 100 | 893 | 25 | | [*Streptomyces viridochromogenes* DSM 40736] | | Protein folding and stabilisation |
| 1297 | BBAL3_51 | | *tldD/ pmbA* | TldD/PmbA family | 0.69 | | 0.03 | **-** | 1.15 | 49.78/28 | | 5.09/5.0 | 96.32 |  | 14 | | [*Brevundimonas sp.* BAL3] | | Protein degradation |
| **Carbon metabolism** | | | | | | | | | | | | | | | | | | | |
| 1461 | SSTG_00732 | |  | glyceraldehyde-3-phosphate dehydrogenase, type I | 0.55 | | 0.08 | - | 1.26 | 36.43/20 | | 5.13/5.9 | 99.92 | 74 | 5 | | [*Streptomyces sp*. e14] | | Glycolysis |
| 652 | SCO3096 | | *eno* | phosphopyruvate hydratase (enolase) | 0.52 | | 0.02 | - | 1.39 | 45.56/43 | | 4.48/4.5 | 100 | 207 | 8 | | [*Streptomyces coelicolor* A3(2)] | | Glycolysis/ Glucogenogenesis |
| 654 | SCO3096 | | *eno* | phosphopyruvate hydratase (enolase) | 0.51 | | 0.01 | - | 1.41 | 45.57/43 | | 4.48/4.5 | 100 | 221 | 9 | | [*Streptomyces coelicolor* A3(2)] | | Glycolysis/ Glucogenogenesis |
| **Amino acids biosynthesis** | | | | | | | | | | | | | | | | | | | |
| 1310 | CV_2767 | | *dhC* | aspartate-semialdehyde dehydrogenase | 0.61 | | 0.13 | **-** | 1.23 | 35.9/28 | | 4.92/5.5 | 98.28 | 58 | 6 | | [*Chromobacterium violaceum* ATCC 12472] | | Amino acids biosynthesis |
| 1229 | SSQG_01989 | | *hisA/ trpF* | bifunctional HisA/TrpF protein | 0.68 | | 0.03 | **-** | 1.14 | 25.56/28 | | 4.76/4.8 | 100 | 451 | 12 | | [*Streptomyces viridochromogenes* DSM 40736] | | Amino acids biosynthesis |
| **DNA metabolism** | | | | | | | | | | | | | | | | | | | |
| 1138 | APM_0032 | | *trwC* | conjugative relaxase domain protein | 0.63 | | 0.09 | **-** | 1.29 | 105.9/35 | | 9.56/6.5 | 98.24 |  | 17 | | [*Acidiphilium sp.* PM] | | Horizontal DNA transfer (site specyfic cleavage of the transfer DNA) |
| 830 | APM_0032 | | *trwC* | conjugative relaxase domain protein | 0.48 | | 0.02 | **-** | 1.54 | 105.9/50 | | 9.56/6.0 | 99.94 |  | 19 | | [*Acidiphilium sp.* PM] | | Horizontal DNA transfer (site specyfic cleavage of the transfer DNA) |
| 1514 | SSQG_03895 | | *ssb* | single-strand binding protein | 0.31 | | 0.02 | **-** | 2.15 | 19.76/25 | | 5.29/5.3 | 100 | 185 | 4 | | [*Streptomyces viridochromogenes* DSM 40736] | | DNA replication repair and recombination |
| **Signalling proteins** | | | | | | | | | | | | | | | | | | | |
| 1376 | SSQG_01951 | |  | two-component system response regulator | 0.51 | | 0.03 | **-** | 1.57 | 24.2/25 | | 4.74/6.0 | 100 | 294 | 10 | | [*Streptomyces viridochromogenes* DSM 40736] | | Signal transduction |
| 1301 | Cbei_3045 | |  | methyl-accepting chemotaxis sensory transducer | 0.66 | | 0.15 | **-** | 1.10 | 62.94/30 | | 4.93/5.5 | 98.2 |  | 15 | | [*Clostridium beijerinckii* NCIMB 8052] | | Signal transduction |
| **Others** | | | | | | | | | | | | | | | | | | | |
| 1464 | Acid345_0176 | |  | radical SAM protein | 0.54 | | 0.16 | **-** | 1.11 | 39.73/20 | | 8.03/5,0 | 98.11 |  | 12 | | [*Candidatus Koribacter versatilis* Ellin345] | | Generation of radical species |
| 726 | BFAG_02012 | | *cobD* | cobalamin biosynthesis protein CobD | 0.82 | | 0.01 | **-** | 1.10 | 36.18/40 | | 8.7/5.1 | 98.88 |  | 12 | | [*Bacteroides fragilis* 3_1_12] | | Aerobic pathway of cobalamin biosynthesis |
| 1255 | SSQG_03772 | |  | uracil phosphoribosyltransferase | 0.64 | | 0.10 | **-** | 1.19 | 23.1/28 | | 4.91/5.0 | 100 | 166 | 10 | | [*Streptomyces viridochromogenes* DSM 40736] | | Nucleotide biosynthesis |
| 799 | SSQG_03670 | | *pstS* | phosphate ABC transporter, phosphate-binding protein PstS | 0.49 | | 0.07 | **+** | 1.36 | 38.67/35 | | 6.01/5.7 | 99.99 | 78 | 6 | | [*Streptomyces viridochromogenes* DSM 40736] | | Transport of phosphate |
| 937 | SSQG_02905 | |  | conserved hypothetical protein | 0.49 | | 0.00 | **-** | 1.67 | 31.1/38 | | 5.01/5.01 | 100 | 199 | 6 | | [*Streptomyces viridochromogenes* DSM 40736] | | Putative F420-dependent oxidoreductase |
| 1469 | SSQG_07592 | |  | secreted protein | 0.43 | | 0.08 | **-** | 1.36 | 61.97/16 | | 9.01/5.0 | 100 | 216 | 4 | | [*Streptomyces viridochromogenes* DSM 40736] | | Putative lysyl oxidase |
| 1435 | SSQG_07014 | |  | secreted protein | 0.34 | | 0.04 | **-** | 1.52 | 19.45/20 | | 4.89/5.0 | 100 | 466 | 6 | | [*Streptomyces viridochromogenes* DSM 40736] | | Putative NADPH dependent riboflavin mononucleotide reductase |
| 1458 | SSQG_07014 | |  | secreted protein | 0.46 | | 0.15 | **-** | 1.20 | 19.45/20 | | 4.89/5.0 | 100 | 182 | 5 | | [*Streptomyces viridochromogenes* DSM 40736] | | Putative NADPH dependent riboflavin mononucleotide reductase |
| 1399 | SSQG_07014 | |  | secreted protein | 0.73 | | 0.00 | **-** | 1.24 | 19.45/22 | | 4.89/5.0 | 100 | 444 | 8 | | [*Streptomyces viridochromogenes* DSM 40736] | | Putative NADPH dependent riboflavin mononucleotide reductase |
| **MacnA, upregulated proteins** | | | |  |  | |  |  |  |  | |  |  |  |  | |  | |  |
| **Protein synthesis and protein turnover** | | | | | | | | | | | | | | | | | | | |
| 947 | SSQG_04757 | |  | translation elongation factor Tu | 1.38 | | 0.06 | **+** | 1.12 | 43.79/55 | | 5,0/5.1 | 100 | 264 | 11 | | [*Streptomyces viridochromogenes* DSM 40736] | | Protein biosynthesis/ Translation |
| 1066 | SSQG_04757 | |  | translation elongation factor Tu | 1.29 | | 0.04 | **+** | 1.10 | 43.79/50 | | 5.0/5.1 | 100 | 883 | 20 | | [*Streptomyces viridochromogenes* DSM 40736] | | Protein biosynthesis/ Translation |
| 1364 | SSQG_04757 | |  | translation elongation factor Tu | 1.55 | | 0.34 | **+** | 1.21 | 43.79/40 | | 5.0/5.0 | 100 | 109 | 4 | | [*Streptomyces viridochromogenes* DSM 40736] | | Protein biosynthesis/ Translation |
| 2402 | SSQG_05040 | | *greA* | transcription elongation factor GreA | 1.37 | | 0.06 | **-** | 1.10 | 17.7/10 | | 4.63/4.8 | 100 | 363 | 9 | | [*Streptomyces viridochromogenes* DSM 40736] | | Protein biosynthesis/ Transcription |
| 1149 | SSQG_05128 | |  | translation-associated GTPase | 1.24 | | 0.06 | **-** | 1.13 | 39.49/40 | | 4.8/4.9 | 100 | 746 | 19 | | [*Streptomyces viridochromogenes* DSM 40736] | | Protein biosynthesis/ Translation |
| 2205 | SSQG_04311 | |  | anti-sigma factor | 1.51 | | 0.10 | **-** | 1.15 | 15.02/23 | | 4.47/4.5 | 100 | 385 | 6 | | [*Streptomyces viridochromogenes* DSM 40736] | | Protein biosynthesis/ Transcription |
| 544 | SSQG_04185 | | *dnaK* | chaperone DnaK | 1.38 | | 0.11 | **-** | 1.14 | 66.19/70 | | 4.76/4.7 | 100 | 397 | 16 | | [*Streptomyces viridochromogenes* DSM 40736] | | Protein folding and stabilisation |
| **Fatty acids metabolism** | | | | | | | | | | | | | | | | | | | |
| 2397 | SSQG_05430 | |  | methylmalonyl-CoA epimerase | 1.90 | | 0.08 | **-** | 1.37 | 16.34/16 | | 4.99/5.1 | 99.99 | 24 | 7 | | [*Streptomyces viridochromogenes* DSM 40736] | | Fatty acids catabolism |
| **Energy metabolism** | | | | | | | | | | | | | | | | | | | |
| 796 | SSQG_05406 | |  | ATP synthase F1, beta subunit | 2.23 | | 0.10 | **-** | 1.36 | 51.97/60 | | 4.78/4.8 | 100 | 99 | 16 | | [*Streptomyces viridochromogenes* DSM 40736] | | Energy metabolism/ATP synthesis |
| **Others** | | | | | | | | | | | | | | | | | | | |
| 1769 | SSQG_01256 | |  | forkhead-associated protein | 1.40 | | 0.05 | **-** | 1.18 | 31.5/33 | | 6.45/5.5 | 100 | 124 | 11 | | [*Streptomyces viridochromogenes* DSM 40736] | | Different cellular processes incl. DNA repair, transcription regulation |
| 2023 | SSQG_01539 | |  | glycerol operon regulatory protein | 1.59 | | 0.11 | **+** | 1.11 | 28.14/28 | | 5.66/5.9 | 100 | 467 | 12 | | [*Streptomyces viridochromogenes* DSM 40736] | | Glycerol biosynthesis |
| 1634 | SSQG_02356 | | *fabD* | malonate decarboxylase, epsilon subunit | 2.34 | | 0.13 | **-** | 1.52 | 32.05/35 | | 5.0/5.0 | 100 | 150 | 5 | | [*Streptomyces viridochromogenes* DSM 40736] | | Malonate biosynthesis |
| 1644 | SSQG_03235 | |  | 4-(cytidine 5'-diphospho)-2-C-methyl-D-erythritol kinase | 1.59 | | 0.06 | **-** | 1.10 | 30.08/35 | | 4.69/4.8 | 100 | 81 | 7 | | [*Streptomyces viridochromogenes* DSM 40736] | | Steroids biosynthesis |
| 2022 | pO86A1_p160 | | *aph* | aminoglycoside-3'-O-phosphotransferase | 1.45 | | 0.02 | **-** | 1.28 | 29.31/25 | | 4.64/4.8 | 100 | 496 | 13 | | [*Escherichia coli*] | | Aminoglycoside resistance |
| 2376 | SSQG_01640 | | *sepF1* | cell division protein SepF1 | 4.97 | | 0.20 | **-** | 1.30 | 15.84/16 | | 4.56/4.5 | 100 | 62 | 9 | | [*Streptomyces viridochromogenes* DSM 40736] | | Cellular growth |
| 1894 | SSQG_01691 | |  | secreted protein | 1.41 | | 0.05 | **-** | 1.15 | 28.56/31 | | 4.76/4.9 | 100 | 216 | 7 | | [*Streptomyces viridochromogenes* DSM 40736] | | Putative sporulation control |
| 2012 | SSQG_02341 | |  | conserved hypothetical protein | 1.63 | | 0.04 | **-** | 1.22 | 17/25 | | 4.26/4.0 | 99 | 80 | 4 | | [*Streptomyces viridochromogenes* DSM 40736] | | Unknown |
| **MacnA, downregulated proteins** | | | |  |  | |  |  |  |  | |  |  |  |  | |  | |  |
| **Protein synthesis and protein turnover** | | | | | | | | | | | | | | | | | | | |
| 916 | SSQG_02509 | |  | glycyl-tRNA synthetase | 0.52 | | 0.04 | **-** | 1.23 | 52.86/50 | | 5.59/5.9 | 100 | 104 | 8 | | [*Streptomyces viridochromogenes* DSM 40736] | | Protein synthesis/ Translation |
| 1979 | SSQG_02132 | |  | serine protease | 0.67 | | 0.10 | **+ +** | 1.16 | 32.9/28 | | 4.94/4.9 | 100 | 406 | 14 | | [*Streptomyces viridochromogenes* DSM 40736] | | Protein degradation |
| **Stress proteins** | | | | | | | | | | | | | | | | | | | |
| 1391 | SSQG_05458 | |  | thioredoxin | 0.53 | | 0.07 | **-** | 1.11 | 34.99/40 | | 4.52/4.5 | 100 | 342 | 12 | | [*Streptomyces viridochromogenes* DSM 40736] | | Oxidative stress response |
| 2158 | SSQG_02339 | | *terZ, terD* | tellurium resistance protein | 0.41 | | 0.00 | **+** | 1.76 | 20.38/25 | | 4.54/4.5 | 100 | 86 | 7 | | [*Streptomyces viridochromogenes* DSM 40736] | | Tellurium resistance/ Stress response |
| 1703 | SSQG_00322 | |  | stress-inducible protein | 0.72 | | 0.03 | **+** | 1.17 | 32.42/33 | | 5.7/5.8 | 99.1 | 66 | 5 | | [*Streptomyces viridochromogenes* DSM 40736] | | Stress response |
| **Fatty acids metabolism** | | | | | | | | | | | | | | | | | | | |
| 2055 | SSQG_01736 | | *fabG* | 3-oxoacyl-[acyl-carrier-protein] reductase | 0.69 | | 0.07 | **-** | 1.19 | 25.78/25 | | 5.33/5.9 | 100 | 245 | 9 | | [*Streptomyces viridochromogenes* DSM 40736] | | Fatty acid biosynthesis |
| **Others** | | | | | | | | | | | | | | | | | | | |
| 1970 | SSQG_02020 | | *sepF2* | cell division protein SepF2 | 0.79 | 0.01 | | **-** | 1.17 | 23.78/30 | | 5.21/5.5 | 100 | 192 | 11 | | [*Streptomyces viridochromogenes* DSM 40736] | | Cellular growth |
| 1701 | HMPREF0873_01794 | | *yhbG* | ABC transporter, ATP-binding family protein | 0.51 | 0.07 | | **-** | 1.10 | 26.4/48 | | 5.47/6.0 | 97.97 |  | 10 | | [*Veillonella sp.* 3_1_44] | | Transport of branched-chain amino acids |
| 1442 | SSQG_06852 | | *idh* | monomeric isocitrate dehydrogenase | 0.44 | 0.16 | | **-** | 1.18 | 79.45/40 | | 4.98/4.9 | 100 | 196 | 10 | | [*Streptomyces viridochromogenes* DSM 40736] | | TCA cycle |
| 1483 | SSQG_05319 | |  | dehydrogenase | 0.75 | 0.03 | | **-** | 1.10 | 34.22/40 | | 5.64/6.0 | 99.99 | 99 | 4 | | [*Streptomyces viridochromogenes* DSM 40736] | | Unknown |
